# Parental considerations during complementary feeding in higher income countries: a systematic review of qualitative evidence

**DOI:** 10.1017/S1368980021001749

**Published:** 2021-04-20

**Authors:** Eleni Spyreli, Michelle C McKinley, Moira Dean

**Affiliations:** 1 Centre for Public Health, Queen’s University Belfast, Institute of Clinical Science, Block A, Grosvenor Road, Belfast BT12 6BA, UK; 2 Institute for Global Food Security, Queen’s University Belfast, Chlorine Gardens, Belfast BT9 5DL, UK

**Keywords:** Systematic review, Complementary feeding, Parental practices

## Abstract

**Objective:**

Worldwide data suggest a clash between parental complementary feeding practices and recommendations. Understanding the circumstances under which parents form their feeding practices is a crucial step to improve such practices. This paper aimed to systematically review the existing qualitative literature and synthesise the factors that parents take into consideration in relation to complementary feeding.

**Design:**

A systematic review was undertaken. Four electronic databases were searched for qualitative studies published after 2001 exploring parental experiences during complementary feeding. A framework that included authors’ outcomes of interest was used to extract and synthesise study findings. The Standards for Reporting Qualitative Research were used to critically assess the included studies.

**Setting:**

Upper-middle- and high-income countries.

**Participants:**

Parents with a child below the age of 3 years.

**Results:**

A total of forty-seven studies met the eligibility criteria and were included in this systematic review. The themes were organised into three main categories: (1) factors related to introduction of complementary foods; (2) factors related to the type of complementary foods and (3) factors related to both timing and type. The selected literature highlights: prevalent baby cues that prompt parents to introduce solid foods; parents’ views on the recommended timing of complementary feeding; factors that drive the choice of complementary foods and perceived value in advice received from health professionals and grandmothers.

**Conclusions:**

This systematic review indicates factors that can be barriers to complying with the complementary feeding guidelines, and therefore, its findings are pertinent to improving parental feeding practices through intervention studies and through infant feeding education in a primary care setting.

In the past two decades, research has considerably advanced our knowledge of infant nutrition by elucidating the rapid physical and neurological development taking place in the first year of life and the consequent nutritional requirements^(1)^. Around the age of 6 months, milk is no longer sufficient to meet infant’s needs in energy, protein, most vitamins and minerals^(2)^, and the addition of complementary foods is required to maintain healthy growth, a process known as complementary feeding^(3)^. Complementary foods are often referred to as solid foods, even though they can be any nutrient-containing foods with a solid, semi-solid or liquid consistency excluding breastmilk or breastmilk substitutes.

In 2001, WHO published a set of infant feeding recommendations advising that infants should be introduced to nutritious complementary foods from 6 months^(4)^. Parents are encouraged to offer a variety of flavours and textures and to include Fe-rich foods, as infant stores in Fe start to diminish from 6 months. It is recommended that added salt is avoided^(5)^, and that parents should abstain from giving sugar- or honey-containing foods^(6)^. In addition, the WHO guidelines advise parents on portion sizes and frequency of meals, on a feeding environment with no distractions, as well as on food safety when preparing and storing food^(7)^. Since the advent of the WHO guidelines, new research evidence has emerged in relation to the appropriate timing of introduction of some foods in children with a risk of food allergy, as well on parental approaches that can help increase food acceptance and encourage children to regulate their food intake^(2,8,9)^. The WHO recommendations, however, remain the basis for complementary feeding guidelines in most countries around the world.

Parental complementary feeding practices show considerable variation across higher income countries and often deviate greatly from the recommendations in terms of the timing of complementary feeding and the nutritional value of the first foods offered. In the UK, the 2011 Diet and Nutrition Survey of Infants and Young Children with a nationally representative sample of 2683 children aged between 4 and 18 months highlighted feeding practices that compromised infants’ dietary quality and diversity^(10)^. Results reveal frequent consumption of nutrient-poor foods (e.g. sugar-containing foods and drinks), along with a low intake of protein- and Fe-rich foods. In South Africa, a cross-sectional analysis of data from 316 children between 4 and 18 months revealed frequent consumption of carbonated drinks, as well as low dietary variety (i.e. consumption of less than 4 food groups daily) and insufficient intake of key micronutrients including Zn and Fe^(11)^. In the USA, the latest Feeding Infants and Toddler Survey shows that in a sample of 902 children between 6 and 12 months, a third of them consumed sugar-containing foods and half of them did not consume protein-rich foods daily (meat, fish, egg)^(12)^. Additionally, the same data indicate that, although rates of early introduction of solid foods are improving through the years, 17 % infants were introduced to complementary foods by 4 months. In Australia, this proportion was 28 % according to the national infant feeding survey in 2010^(13)^. In Brazil, a prospective study which assessed feeding practices and dietary intake of 179 healthy infants showed that the median age of introducing solid foods was 4 months and concluded that insufficient intake in both macro- and micronutrients in young infants was very frequent with 45 % being Fe deficient^(14)^. Introducing solid foods early has been shown to be prevalent in European countries too^(15,16)^.

Understanding the circumstances under which parents form their feeding practices, particularly practices that are in conflict with the recommendations, is a crucial initial step to improve them. Soaring interest has therefore focused on utilising qualitative methodologies^(17)^, which have been useful in providing insights into how and why parents shape their feeding practices. In the past two decades, new evidence exploring parental experiences in relation to the introduction of solid foods has emerged, and two systematic reviews have provided syntheses of this evidence^(18,19)^. However, given the ever-growing number of relevant qualitative studies published since the last systematic review, it was considered essential to cast a view in the literature to gain a greater understanding of the underlying factors that contribute to the formation of parents’ feeding practices and their adherence to complementary feeding guidelines. In doing so, attention was drawn to the fact that although the WHO guidelines are relevant for all countries across the globe, circumstances in low-income countries (e.g. high rates of child stunting, insufficient health information services and limited access to clean water) represent unique and distinct challenges for infant feeding, when compared with higher income countries^(20)^. This paper aimed to systematically review the existing qualitative literature in order to synthesise all the factors that are taken into consideration by parents and primary caregivers in relation to complementary feeding in upper-middle-income and high-income countries. Findings of this review will enable the design and implementation of effective education initiatives that will encourage parent feeding practices in line with complementary feeding recommendations.

## Methods

The protocol of this review has been registered in the PROSPERO database (registration ID: CRD42017067091). The selection of the reviewed studies was based on a number of eligibility criteria detailed in Table 1. The studies that satisfied the eligibility criteria were incorporated into the synthesis; no further considerations were included (e.g. data saturation).

**Table 1:** Eligibility criteria for inclusion and exclusion of reviewed studies

Participants	Parents or primary caregivers of healthy infants (from birth to 3 years of age). Studies including children up to 3 years of age and older were excluded, unless they reported results separately for different age groups.
Study design	Studies of qualitative methodology as well as mixed-method studies with a significant qualitative component. Qualitative research related to an intervention was excluded.
Setting	Studies that interviewed participants residing in ‘upper-middle-income’ and ‘high-income’ economies as per the World Bank Classification (1). Challenges observed in lower-income countries regarding infant feeding represent unique and distinct challenges that require separate focus, and therefore, relevant studies were excluded.
Objective	Qualitative research that discusses the process of complementary feeding, including introduction of solid foods as well as further stages of familiarisation with family diet. Studies that solely explored experiences regarding breast-feeding or cessation of breast-feeding with no reference to complementary foods were excluded.
Year of publication	Studies that were published or completed data collection from 2001, following publication of WHO complementary feeding guidelines (2). This criterion was established, as it was within the remit of authors’ interest to extract parental attitudes towards the guidelines.

### Information sources and search

Four electronic databases were searched for this review: PubMed, EMBASE, Scopus and Web of Science. The papers retrieved from all databases were published in the same reference period (January 2001 – 6 March 2021). The reference lists of all included studies were hand-searched for additional papers that meet the inclusion criteria. In a few cases, where a full text could not be retrieved in English, the authors of these papers were contacted to confirm that a paper in English did not exist.

The same terms were used to search all databases after appropriate adjustment for database-specific operators. A list of search terms can be found in Table 2. Limits for the exclusion of papers published before January 2001 and animal studies were applied when possible. No limit was applied for language of publication.

**Table 2:** Keywords used in search of electronic databases

1.	Weaning NOT ventilation
2.	Infant feed*
3.	Complementary food*
4.	Complementary feed*
5.	“Solid food introduction”
6.	1. OR 2. OR 3. OR 4. OR 5.
7.	“Information sources”
8.	Knowledge
9.	Attitude*
10.	Practice*
11.	7. OR 8. OR 9. OR 10.
12.	6. AND 11.

The asterisk (*) was used as the wildcard character in the search.

### Study selection

All search results were imported from each database to Refworks citation management software (ProQuest). They were then exported into a unified Excel spreadsheet. All duplicate records were identified and removed. Titles were screened, and the records with irrelevant titles were removed. Two reviewers independently reviewed all abstracts of the remaining records against the eligibility criteria (ES, MMK). Disagreements were due to simple oversights and were resolved through discussion. When a definite decision could not be made based on the studies’ abstract alone, the full paper was obtained for a thorough assessment against the inclusion criteria. The reasons for exclusion were documented for each study that was screened on a full-text basis.

### Data collection and synthesis

The information that was obtained and integrated in this review included key characteristics and reported findings of the included papers. In order to extract and synthesise study findings, a framework approach was followed as described by Barnett-Pate and Thomas^(21)^. An initial framework (i.e. a list of themes) was conceived following review of the background literature and discussions among authors. After selection of relevant studies and during careful consideration of the available data, additional themes emerged and incorporated into the initial framework. However, during this process, the newly emerging themes were prioritised based on frequency of occurrence within the selected literature. In this way, themes that appeared in only few papers and that were not extensively discussed were not presented in this review, an approach named as Qualitative Meta-summary^(22)^.

### Quality assessment in individual studies

The ‘Standards for Reporting Qualitative Research’ tool (SRQR) was used to assess the quality of the included studies^(23)^. The SRQR checklist includes twenty-one criteria and provides a critical appraisal of the design and reporting of findings for the eligible papers. The SRQR scoring was utilised to underline areas of methodological rigor and clarity of reporting, as well as key elements that were inadequately reported. In this way, the quality assessment of the selected studies could enable comparisons among them and inform qualitative researchers of common omissions. Studies were not excluded based on the quality assessment, and their scores were not taken into consideration during data synthesis.

## Results

### Study selection

**Fig. 1:**
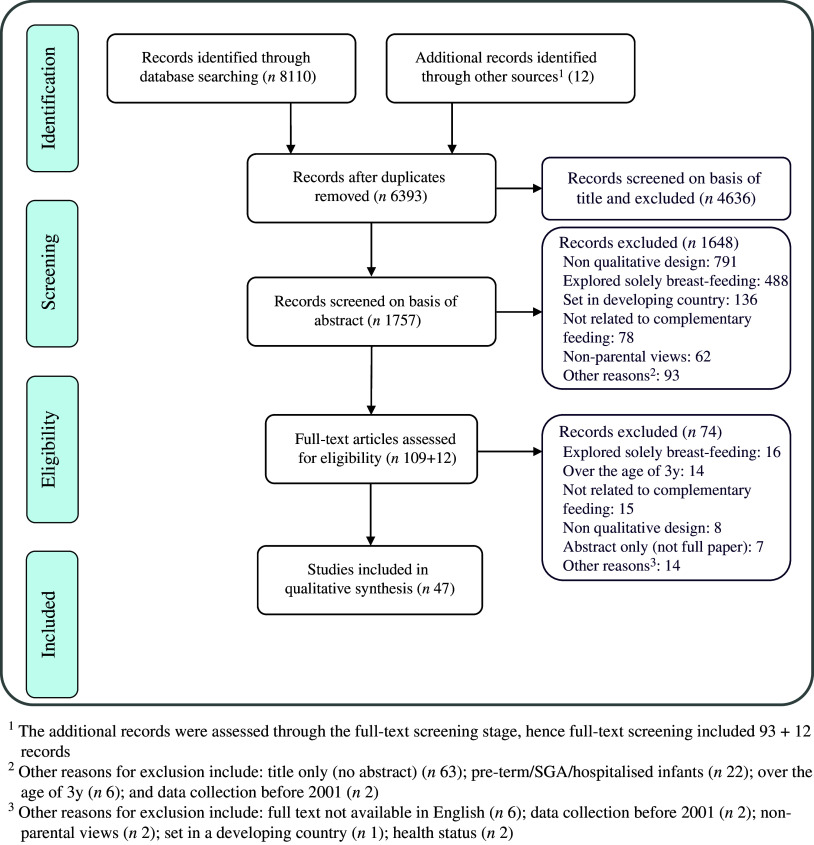
PRISMA flowchart of study selection

Figure 1 provides a flowchart of the selection process based on the PRISMA protocol^(24)^. Forty-seven studies were eligible and considered for this review. Characteristics of each of these studies can be found in Appendix – Table 1. Additionally, a list of all studies that were reviewed on a full-text basis and were not included is provided in Appendix – Table 2 with the reason for exclusion.

### Quality assessment of included studies

**Fig. 2:**
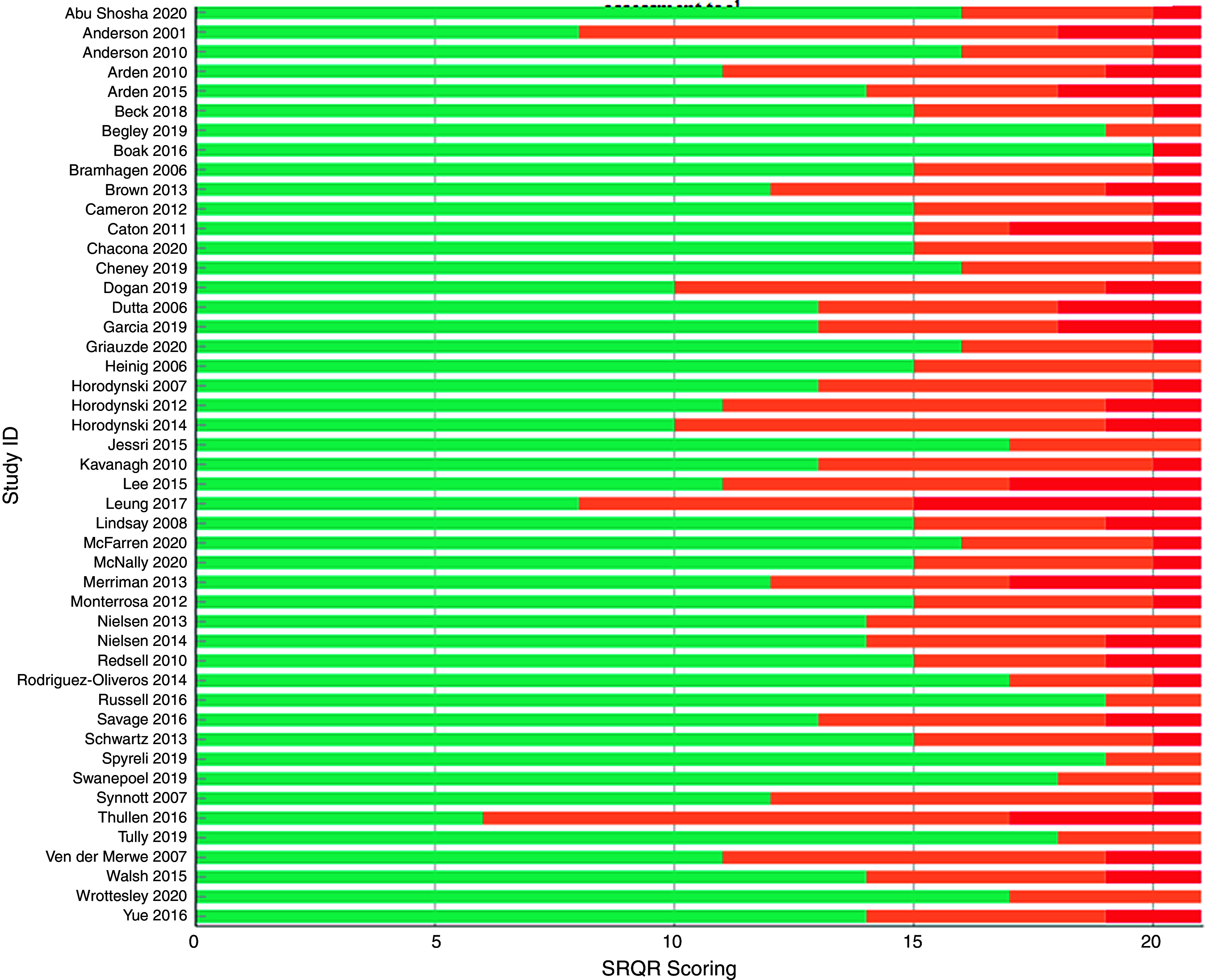
Scoring of individual studies according to the ‘Standards of Reporting Qualitative Research’ assessment tool. 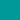, Number of items sufficiently covered in paper; 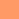, number of items partially covered in paper; 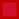, number of items for which no information was provided

All the manuscripts were evaluated against the twenty-one items of the SRQR checklist (Fig. 2). All included studies sufficiently reported the problem formulation and the research question. All but one^(25)^ adequately covered their results and their interpretation. The data analysis process was not well reported in five studies. Links to empirical data (e.g. quotes) were reported in 91 % of studies (43/47) and source of funding in 89 % of studies (42/47).

Incomplete information was apparent within the included papers for a number of areas, one of which was describing the methodological approach followed within the title. Furthermore, there was a lack of information on the level of participation in each study and why. Finally, in twenty-four studies there was insufficient integration with prior work and inadequate descriptions of the transferability of studies’ conclusions and contribution to the field. The most frequently omitted information was regarding authors’ conflict of interest, followed by techniques enhancing credibility and study limitations. A detailed comparison of the studies against the SRQR checklist is provided in Appendix – Table 3.

### Study findings

**Fig. 3:**
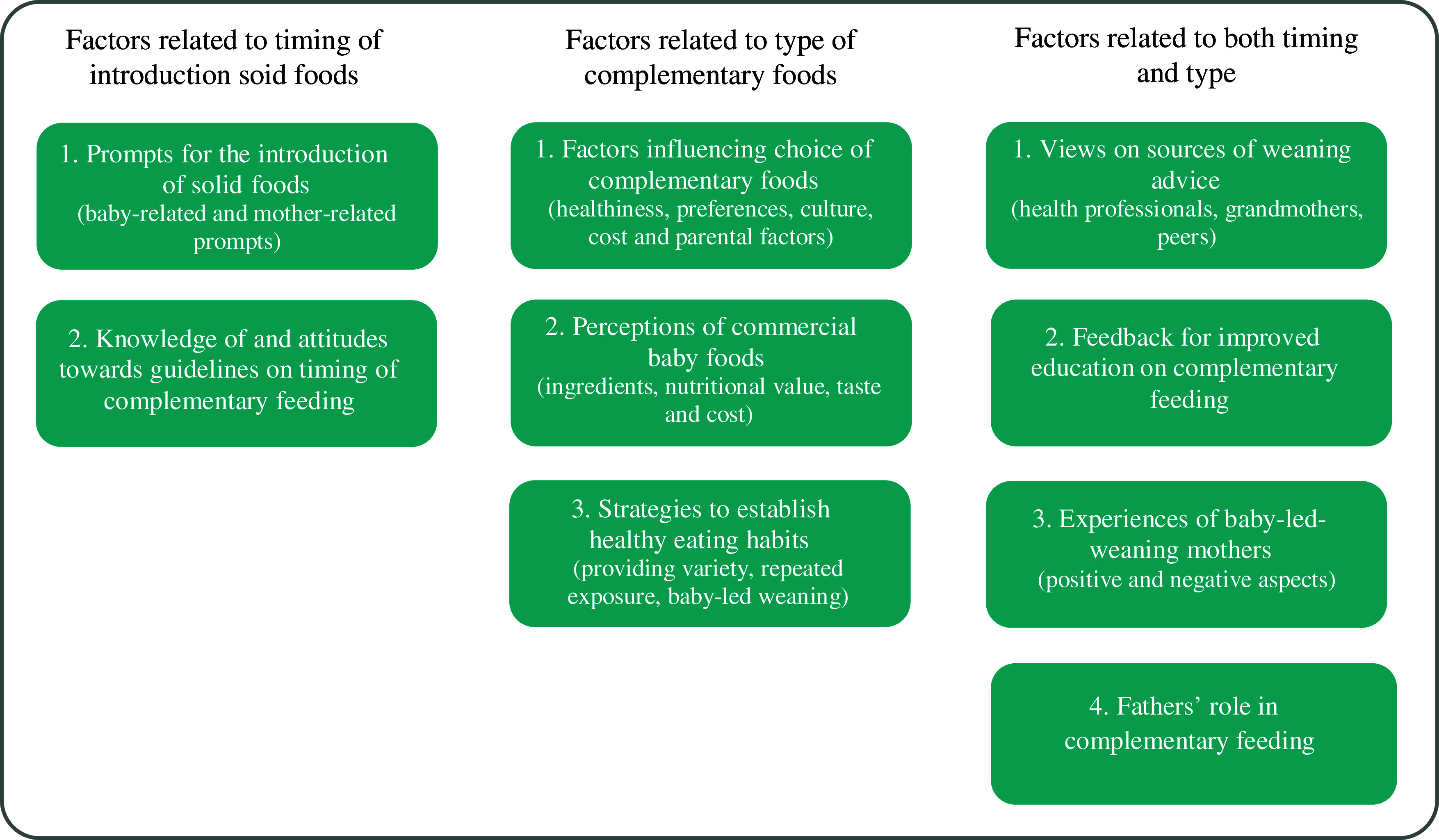
Themes and sub-themes emerging from reviewing the qualitative research on complementary feeding. Themes are classified into (i) factors related to the timing of complementary feeding; (ii) factors related to the type of complementary foods and (iii) factors related to both timing and type of complementary foods

The themes were classified into (i) factors related to the timing of complementary feeding; (ii) factors related to the type of complementary foods and (iii) factors related to both timing and type of complementary foods. Figure 3 shows in detail the classification of themes and sub-themes discussed under each category. The themes were addressed across the literature with the majority of included papers discussing all themes. The frequency with which the themes and sub-themes were reported is summarised in Appendix – Table 4.

### Factors related to timing of introduction of solid foods

#### Prompts for the introduction of solid foods

Prompts were infant- and mother-related.

##### Baby-related prompts

Mothers discussed how cues from their babies guided them to introduce solid foods. These cues included perceived hunger, perceived interest in food, changes in weight and physical cues indicating their infant’s readiness for solids.

The idea that infants were no longer satisfied by a milk-only diet was often cited by mothers and fathers as a compelling cue to give the baby’s first solid foods^(25–44)^. Signs that indicated hunger were crying, increased consumption of milk and disturbed sleep. In some cases, the feeling of obligation to attend to baby’s hunger led parents to introduce solids earlier than indicated by their country’s guidelines^(25,29,33–36,43)^. Seeing baby settle or sleep longer after providing food justified early complementary feeding.

Excitement triggered by the sight of food was reported as another sign of baby’s readiness for solids^(26–29,31,36,38–40,42,45–48)^. Parents described that their baby was reaching out for food and grabbing it from their plates, which encouraged them to start complementary feeding. A refusal for the breast, as reported in the study by Abu Shosha *et al.*, also urged mothers to introduce solids^(41)^.

Baby’s weight was also discussed in relation to the timing of introduction of solid foods^(26,28,29,32,33,39–42,49)^. The purpose of complementary feeding was quoted to sustain a steady growth rate for babies with slow weight gain^(28,29,33,40,42,49)^. On the other hand, infants whose weight picked up in the first months of life were perceived to need solid foods earlier than their lighter peers^(26,39,41)^.

Mothers explained that a number of physical milestones signified physiological maturation for a smooth transition to a mixed diet, such as losing the tongue thrust, being able to sit up, good hand-eye coordination and teething^(26,28–30,36,42,45,46,48)^.

##### Mother-related prompts

Certain mother-related considerations also contributed to the decision to introduce complementary foods^(26,28,39–41,44,49–51)^. Mothers who breastfed conveyed the need for babies to be fed by other people too, so that they could return to work^(28,39,41,44,50,51)^. Additionally, some mothers shared that they were excited to see their children eat family foods^(26,40)^. One study highlighted that breast-feeding in public was felt as shameful for Middle Eastern mothers living in Canada^(49)^ and introducing solid foods was seen as the solution.

#### Knowledge of and attitudes towards guidelines on timing of complementary feeding

Parental views on the recommended age for introducing solid foods was an important theme across papers in this review.

##### Awareness of the recommended age of complementary feeding

Even though the recommended age of complementary feeding varied slightly based on location (e.g. after 4 months in the USA and from 6 months in the UK and Australia), findings from the selected studies reflected good awareness of the guidance^(26,30,35,38,40,43,45,46)^. In contrast, few studies revealed poor awareness of the appropriate time to start complementary feeding^(41,52–54)^. Indicatively, mothers interviewed by Abu Shosha *et al.* and Yue *et al.* believed that milk is sufficient for the first year of life and hence the introduction of solids foods is not necessary^(41,54)^.

##### Attitudes towards the guidelines for the appropriate age for complementary feeding

A wide range of views was expressed^(26,28,30,34–40,42,43,45–47,55,56)^. There were mothers who questioned their credibility and disregarded them, those who were sceptical but still considered them as a guide and those who expressed an effort and desire to follow them. Some of the reasons of dissatisfaction quoted were that current guidelines are perceived as too rigid and that they put unrealistic restrictions on a new mother. In addition, a ‘one-size-fits-all’ approach of the guidelines is often not viewed as relevant, since all babies develop differently.

##### Understanding the rationale

Despite mothers’ good awareness of the recommended age for complementary feeding, mothers revealed an uncertainty about the health implications of early introduction of solid foods^(26,35)^ and about the need for reviewing and updating the guidelines^(39,42,55)^.

### Factors related to type of complementary foods

#### Factors that influence the choice of complementary foods

Factors taken into consideration by parents to determine the type of complementary foods included health properties of the food; infants’ food preferences; culture; cost and parental considerations.

##### Actual and perceived health properties of the food

Providing a healthy diet was voiced as a priority in most studies that addressed the choice of complementary foods^(26,31,33–35,37–41,43–46,48,50,53–64)^. Parents shared a desire to provide a balanced diet with foods from all groups that will secure a sufficient intake of nutrients. Encouraging fruit and vegetable consumption was often discussed as the foundation of providing a healthy diet. A preference for organic foods was expressed by parents in a number of studies^(34,37–39,44,55,56,61)^, even though some admitted that cost was considerably higher than conventionally sourced foods.

On the contrary, certain foods were perceived as ‘unhealthy’ due to their nutritional content (e.g. sugar and salt) and level of processing and were avoided^(37,45,53,56,57,61,64)^. Parents also discussed complementary feeding as an opportunity for the family to adopt a healthier diet that is more suitable for the child’s nutritional needs. However, offering unsuitable solid foods, such as sugar-sweetened drinks and snacks, was discussed in some of the selected studies^(48,49,59,65,66)^.

Furthermore, foods were avoided for complementary feeding because parents believed that they would cause allergy (e.g. dairy, nuts, eggs and fish)^(37,40,43,50)^ or that an infants’ gastrointestinal system was not mature enough to process them (pulses, meat and some types of vegetables)^(31,37,48,50,57)^. Beck and Yue reported that parents waited for children to reach 12 months before introducing meat, as they believed their infants could not digest it. Gaps in parental understanding of what healthy diet means were evident, but also in the nutritional content of foods and in the nutrient-health links. In some cases, individual foods have been arbitrarily linked to healthy growth and well-being due to their nutritional content^(48,50,60,63)^.

##### Baby’s preferences and aversions

Parents observed that their infants had distinct food likes and dislikes which guided the choice of complementary foods^(26,39,42,45,48,50,54,56–58,60,62)^. Rodriguez *et al.* and Yue *et al.* report that parents might have even prioritised offering foods that baby was thought to like over others that were considered to be more nutritious.

##### Cultural background

Studies explored the values of mothers whose culture is strongly linked to dietary choices and traditional foods automatically featured in infant diet^(33,37,48,50,52,57,58,63,64,66)^. They also highlighted experiences whereby religion was associated with food^(32,49,67)^, such as Islamic beliefs requiring halal complementary foods^(49)^. Some studies discussed how cultural traditions encourage the consumption of foods that are not recommended during infancy, i.e. herbal infusions and sugar-sweetened desserts^(49,52,63,64)^.

##### Food cost

For low-income families, the price of certain foods (fresh fruit, vegetables and meat) was a barrier to providing a complete nutrition during complementary feeding^(34,37,38,48,49,54,58,60,63)^. Due to the reported high cost and perishability of these foods, parents described replacing them by cheaper alternatives of poorer nutritional quality or offering them less frequently than ideally (e.g. daily). The cost of nutritional supplements was an additional financial burden for families with limited budget^(49)^.

##### Parental considerations

Additional factors that guided the decision on the type of first foods were food consistency, parents’ own food preferences and cooking skills^(26,31,34,37–39,42,46,48–50,58,61–64,68)^. Foods with a soft texture were considered appropriate for infants, whereas parents discussed how hard foods, or foods with seeds raised concerns of choking and were, therefore, avoided^(26,37,48,50)^. Food choices were also influenced by what mothers saw as tasty and discussed how infants are exposed to food they preferred^(31,37,46,48,58,61,64)^. Similarly, foods mothers disliked were not available and were not provided to children^(50,62)^. Limited confidence in cooking and lack of cooking equipment were also quoted to impact choice of complementary foods^(34,42,49,50,58,60,63)^.

#### Perceptions of commercial baby foods

A general preference for home-made foods over ready-prepared baby foods emerged from the studies included. Even though commercial foods were seen in some cases as convenient and time-saving^(37–39,44,56,63)^, mothers voiced mistrust for products marketed for infants, because their label ‘suitable from 4 months’ contradicted the 6-month recommendation and could contribute to maternal confusion over timing of complementary feeding^(38–40,42)^. Other negative aspects of marketed baby foods, as perceived by parents, included uncertainty regarding presence of potentially harmful ingredients and nutritional value, inferior flavour and cost.

##### Ingredients and nutritional value

A common idea about ready-prepared baby foods was that they underwent processing and contained unknown ingredients or artificial additives that could be dangerous for infant’s health^(42,44,50,57,58,60,61)^. Their nutritional quality was an additional consideration, and parents were uncertain whether commercial baby foods provided the necessary nutrients and whether they were void from ingredients that should be avoided during infancy, e.g. salt and sugar^(38,39,50,55,56,60)^.

##### Taste and cost

Four studies reported that ready-made infant foods were perceived to lack taste and therefore children did not enjoy eating them^(37,38,55,66)^. In addition, parents felt it was cheaper to make their own baby foods at home than to buy those on the market^(49,58,60,66)^.

#### Parental strategies to establish healthy dietary behaviours

Parents talked about the positive effects of offering a variety of foods, the repeated exposure to foods, baby-led weaning and modelling with regard to future food habits.

##### Providing variety of foods

Mothers were mindful that their feeding approaches would have a lasting effect on their children’s eating behaviours and that ensuring food variety would have a number of benefits, like nutritional adequacy, improved acceptance of foods in infancy and later in life and low risk of fussy eating behaviours^(26,28,37,46,48,53,55,56,58,61,62)^.

##### Repeated exposure to foods

In a number of studies, mothers discussed how their infants’ acceptance of unfamiliar foods grew over time through their repeated exposure to them^(37,55,57,58,62)^. Mothers recounted cases of re-offering foods disliked by their infants (mostly vegetables) like re-presenting them after a few days, hiding them within dishes or giving them side by side with a previously liked food. A coercive repeated exposure was presented by Wrottesley and team, whereby mothers reported force-feeding foods infants refused to eat^(64)^.

##### Baby-led weaning

Six studies discussed baby-led weaning (BLW) (i.e. infant being offered foods in their whole form and self-feeding rather than being spoon-fed^(69)^ as another method to foster healthy eating habits in the future), three of which exclusively recruited BLW mothers in the UK and New Zealand^(28,40,44–47)^. Through this approach, it was envisaged that children would develop an ability to develop good appetite control, lower their chances of becoming fussy-eaters and improved the experience of family meals.

##### Modelling

Mothers considered themselves role models for their children and used this quality in an effort to shape good food habits^(38,55,62)^. This was achieved by eating healthy, nutritious foods (e.g. vegetables) and abstaining from unsuitable snacks (e.g. sweets) in shared mealtimes.

### Factors related to both timing of introduction and type of solid foods

#### Views on the available sources of feeding advice

Discussions in relation to sources of influence on parents’ feeding practices were a common thread in the included studies. Advice from ‘important others’ (healthcare professionals, grandmothers and peers) was described as a prompt to introduce complementary foods and also a guide to the choice of foods offered to their children.

##### Healthcare professionals

A range of professionals who give postpartum feeding advice were the most-frequently-discussed source of information. These ranged from paediatricians and nurses (European countries) to health visitors (UK) and other healthcare professionals (e.g. Special Supplemental Nutrition Program staff in USA)^(25–29,36,41,43,45,47,52,53,55,59,64,67,70)^.

In nine studies, medical professionals were discussed in a positive light due to their experience and knowledge and parents recognised that their decision on when to start complementary feeding and what foods to feed was based on their advice^(26,28,29,38,40,41,47,48,53,55,67,70)^. However, in eleven studies the information from healthcare professionals was met with scepticism^(25,27–31,34–36,39,43,45,64)^. Two separate samples of mothers and fathers agreed that advice from medical staff who had not experienced parenthood was less valued than those with children^(27,39,40)^. The level of adherence or scepticism towards advice from healthcare staff seemed to depend on the nature of contact with them and number of previous children. For instance, Yue *et al.* explored a setting where caregivers expressed mistrust for the advice of medical professionals, but had limited opportunities to consult them due to the absence of a routine postnatal care pathway^(54)^. Moreover, a sample of Danish mothers who had previous experience with complementary feeding were less willing to consult with a health provider^(56)^.

##### Grandmothers

Parents in many studies expressed trust for their mothers’ feeding advice, and in some cases, grandmothers were a preferred source of advice over healthcare professionals^(25,30,31,33,39,41,42,44,49,54,55,59,64,65,71)^. Mothers-in-law were also influential, though to a lesser extent, and particularly for participants who viewed them as a ‘motherly figure’^(26,27,31,35,59,61)^. Perceived value in grandmothers’ advice was due to their own experience with feeding children^(31,41,44,55,59,64,65)^. Teenage mums and women living in a different country from their home country relied heavily on their mothers’ advice which shaped their infant feeding practices^(32,49,65)^. On the other hand, parents reported that the advice from their mothers often came without being requested, as it was considered outdated^(27,28,35,38,39,41)^. It was often pointed out that grandmothers applied pressure for an early introduction of solids^(25,26,35,40,59,61,64)^ and offered or encouraged foods that were not suitable for infants, e.g. sweet treats^(25,34,44,49,58,59,61)^.

##### Peer influence

Friends and peers acted as an important influence and contributed both positively and negatively to parents’ complementary feeding decisions^(25,27–31,34,35,37,39–44,47,49,52,54,55,58,65,70,71)^. Both mothers and fathers highlighted that parenthood and recent experience with infant feeding qualified friends as credible sources of advice^(27,40,58)^. The ease of getting in touch with them and their experience in dealing with practical difficulties of feeding contributed to the parental trust in peers’ advice. It was recognised, however, that the influence of peers sometimes manifested as peer pressure to introduce solid foods early^(28,34,40,42)^.

Receiving external advice was very often expressed as vague, contradictory and confusing^(25,28,31,35–42,49,52,54,58,71)^. The plethora of available sources of information made it difficult for parents to filter out the unreliable information. In general, parents who had previous experience with complementary feeding felt they were less influenced by external advice or had a better ability to gauge if the advice was correct^(27,30,38,40,41,44,52,54)^. Receiving conflicting complementary feeding advice was quoted as a rationale for not adhering to infant feeding guidelines^(28,36,40)^.

#### Feedback from mothers on improved complementary feeding education

Parents suggested improvements for areas where they felt they needed more information or support, as well as healthcare professionals’ approach to providing feeding information.

##### Areas of inadequate information

In several studies, mothers pointed out that early introduction of solid foods is prevalent, as they felt there is insufficient education on its health implications and on the benefits of waiting until 6 months^(25,28,30)^. Furthermore, it was felt that more information was required on the specific foods that are associated with increased risk of allergies^(30,39,65)^. Other areas where more information was required included the appropriate frequency and size of infant’s daily meals^(25,30,37,41,43,53,66)^, as new mothers found it difficult to conceptualise the amount of solid foods that would replace milk feeds. More guidance was also sought after in relation to the appropriate age of introducing different food textures^(41,42)^. Finally, in two recent studies, social media were suggested as an accessible way to communicate infant feeding information to younger mothers^(43,70)^.

##### Healthcare professionals’ role

The ability to establish good rapport was seen as an essential quality that health professionals that deal with parents should have. Participants explained that healthcare staff need to be approachable, inspire trust and avoid passing judgement for mothers’ feeding practices^(34,39,41,44,65)^. In terms of language used, parents highlighted the need for medical staff to translate the dietary guidelines into easy-to-understand practical information^(58,65)^. Participants in studies by Jessri *et al.* and Horodynski *et al.* stressed the importance of receiving complementary feeding advice that is sensitive and appropriate to their ethnic identity and religion^(49,65)^. Receiving information that does not take into consideration mothers’ cultural background was described as an important barrier to adhering to the complementary feeding recommendations^(49)^. Finally, mothers in the UK voiced disillusion with advice from healthcare professionals that were not in alignment with the updated feeding recommendations^(43)^.

#### Experiences of baby-led-weaning mothers

Three of the included studies set out to explore the experiences of mothers who employed a baby-led approach to complementary feeding^(45–47)^. Among BLW mothers, it was generally agreed that the infant feeding guidelines were an important guide that prompted them to wait for 6 months to introduce solid foods. The studies, two from the UK^(45,46)^ and one from New Zealand^(47)^, dealt with the advantages of the method, as well as the practical difficulties.

##### Positive experiences

Good appetite and portion control was described by mothers as one of the advantages over traditional feeding methods. According to BLW mums, their children developed the ability to recognise their own signals of hunger and satiety and demonstrated good control of the amount of food needed to satisfy their hunger^(45,46)^. Additionally, McNally and colleagues observed a reduced maternal concern over the amount of food eaten by BLW infants and whether it is enough, when compared with spoon-feeding mothers^(51)^. Participants in the BLW studies viewed spoon-feeding as a forceful process that was associated with a certain level of pressure, whereas BLW was viewed as a more fun way of eating^(45,46)^. With baby-led weaning, infants were seen to actively take part in family mealtimes and overall made feeding a more enjoyable experience for them and their parents^(44,46,47)^. Finally, some mothers who used BLW explained that the approach saved money and time, as infants have whatever the family eats with no need for particular adaptations such as preparation of purees^(46,47)^.

##### Negative aspects

Mothers admitted that BLW was not suitable for all times and occasions, and the method was linked with increased mealtime mess^(45–47)^. However, mothers discussed that mess was gradually reduced as infant motor skills improved. Although some mothers described BLW as economical, others described the method as wasteful due to the mess made, and this could discourage mothers from offering expensive foods^(46)^. Allowing infants to have control over the food consumed was not always discussed positively, and some mothers were unable to measure the amount eaten which raised concerns around the adequacy of child’s food intake^(45)^. Finally, although mothers admitted that the risk of choking might put parents off of BLW^(43,44,46,47)^, only a small minority reported dealing with individual cases of choking which were eventually managed easily.

#### Fathers’ role in complementary feeding

Two of the included studies set out to explore fathers’ experiences in relation to complementary feeding^(27,68)^. Specifically, Anderson and colleagues recruited exclusively male participants, and Thullen *et al.* recruited couples with both males and females being present during each couple’s interview. Two additional studies included fathers in their sample^(61,71)^ and another two recruited an exclusively female sample, but included mothers’ reports on their male partners’ involvement with complementary feeding^(42,64)^.

Overall, fathers’ role was described to be supportive to a great extent, since mothers were mainly responsible for feeding and took decisions regarding the timing and the type of food^(27,42,64,68,71)^. Moreover, it was highlighted that fathers had a more relaxed approach to feeding and were more inclined towards learning through experience rather than implementing information previously sought from important sources. Fathers reported to be less concerned about feeding their child foods that mothers would consider not appropriate for infants^(61,68)^. Additionally, a sample of fathers described offering specific portions, pre-determined by their partners, rather than responding to infant’s satiety cues^(27)^.

## Discussion

This systematic review offers a comprehensive synthesis of the qualitative evidence in relation to factors that parents take into consideration during complementary feeding in higher income countries. Findings of this paper provide novel insights into parental experiences and attitudes and can be grouped into three overarching categories: (1) themes that relate to the age of introduction of solids foods; (2) themes that relate to the type of first solid foods and (3) themes that relate to both age of introduction and type. Themes with important public health implications include prevalent baby-driven cues to introduce solids; parental views on the recommendations for the age of complementary feeding; factors that drive the choice of complementary foods; perceived value in advice received from health professionals and grandmothers and the nature of fathers’ role during complementary feeding. The findings and their public health implications are discussed in detail below.

### Factors in relation to the age of introduction of solids

Studies included in this systematic review indicated that the circumstances that prompt the introduction of solid foods have been extensively explored among parents from various socio-economic backgrounds. Perceived infant hunger made mothers think that milk alone fails to nourish their babies and was reported to be a compelling prompt for mothers to introduce solids. This has been previously reported in two previous systematic reviews on parental infant feeding practices^(18,19)^. It is also consistent with cross-sectional data, as the UK Diet and Nutrition Survey for Infants and Young Children revealed that the most prevalent reason for introducing solid foods was the perception that the baby is no longer satisfied with milk feeds^(10)^. One of the signs indicating infant hunger, as reported in this review, was disrupted sleep. Contrary to common maternal beliefs, an observational study has shown that increasing the calorific content of infants’ diet by offering more milk or by commencing complementary foods does not improve disrupted sleep^(72)^. Parents need to be more aware of official guidance that warns that hunger and lack of sleep alone do not mark an appropriate timing for introducing solids and encourages them to be mindful of developmental milestones too^(73,74)^. Moreover, the qualitative literature, as reviewed in this paper and in the review by Harrison *et al.* ^(18)^, has demonstrated that having a heavy infant can encourage early introduction of solids, which is in agreement with data from a longitudinal study by Rogers and Blisset^(75)^. Recent research has also addressed infant temperament as a potential contributing factor to the timing of introduction of solid foods, but with no conclusive evidence so far^(75–77)^. In this review, although not explicitly discussed, mothers did talk about every baby being different indicating awareness of baby’s personality. It will be interesting for future qualitative researchers to explore whether parents are conscious of introducing solids earlier or later because of their child’s temperament.

In relation to parental perceptions of the recommendations on the appropriate age for complementary feeding, there was good awareness, but there was scepticism as to whether introducing solids at an age specified by universal guidelines is best practice; this has formerly been demonstrated by the systematic reviews on this topic as well^(18,19)^. In addition, findings indicate that there was a substantial gap in maternal knowledge of the health risks related to early introduction and similarly the benefits of timely introduction, which seem to reinforce the general dismissal of the recommended timing for complementary feeding. Poor compliance to the recommendation on timing of complementary feeding is prevalent across higher income countries and is illustrated by national survey data in the USA, Australia and UK, where the officially recommended timing of complementary feeding is 6 months^(13,78,79)^.

### Factors in relation to the type of first solid foods

Foods’ nutritional content and their impact on infant’s health were the most influential factors when choosing complementary foods. Placing emphasis on offering fruit and vegetables was seen as an important determinant of choice of complementary foods, and this was shown by previous systematic reviews in this topic^(18,19)^. Indeed, the value of introducing a wide variety of fruits and vegetables early in life has been highlighted in previous research^(8)^. Furthermore, purchasing organic was seen in some cases as a way of securing the best possible diet for their children. In contrast to the beliefs of many parents, the latest systematic review on the nutritional quality of organic foods by Dangour and colleagues concluded that currently there is no evidence suggesting significant differences in the nutrient content between organic and conventionally produced food^(80)^.

Among the factors that influenced the type of complementary foods offered, discussions revealed parental concerns regarding the consumption of certain foods during the first years of life. Some of these concerns were passed down from older family members or were established on an empirical basis. Fear of developing food allergies was quoted as another reason to avoid a number of foods. Similarly, in the most recent UK Infant Feeding Survey, 12 % and 11 % of mums with an infant aged between 8 and 10 months reported that they avoided giving to their children any eggs or dairy products, respectively, due to fear of allergy^(79)^. Food avoidance can restrict nutritional variety and compromise nutrient sufficiency^(81)^. Therefore, current findings in conjunction with previous observations regarding food avoidance indicate that parents can benefit from receiving clear and evidence-based advice on food transitioning during infancy, as well as advice on the risk of food allergy based on family history by health professionals with relevant expertise.

### Factors in relation to both age of introduction and type of solid foods

Among a number of sources of advice on complementary feeding, grandmothers and health professionals generated most discussion. Findings showed that views on the information given by medical professionals were polarised. Their advice was better received when it was incorporated through scheduled visits relative to other settings where caregivers had limited opportunities to consult a health professional. Another contributing factor of the diverging views might be the varying levels of health professionals’ knowledge of current evidence on complementary feeding. A survey by Chouraqui *et al.* revealed a discordance between parents’ statements of the infant feeding advice received and the statements of paediatricians who offered this advice^(82)^. Hence, an assessment of healthcare staff’s knowledge of current complementary feeding guidelines and their perceived barriers to an effective nutritional education is required to complement parental perspectives and give directions for improved access to feeding recommendations. When it came to parental perspectives on grandmothers’ advice, mothers with great respect for older generations and teenage mums put a lot of trust in their mothers’ advice. However, such advice can be regarded as outdated and diverging from the official complementary feeding guidelines. Cross-sectional data confirm that infant feeding advice received by grandmothers can be undermining of official feeding guidelines by applying pressure to add cereal in the infant’s bottle^(83)^. Adolescent mothers have been shown to be particularly influenced by their mothers’ advice, as they are more likely to live and have daily contact with them compared with adult mums^(84)^. Furthermore, findings from observational studies suggest that grandmothers may have a negative influence on breast-feeding duration, but support provided by healthcare professionals has been associated with longer breast-feeding duration and postponed introduction of solid foods^(85–88)^. Therefore, including grandmothers in counselling sessions with health professionals on complementary feeding could enhance grandmothers’ knowledge and support of recommended feeding practices. More studies are needed in order to assess the potential benefits and negative effects of external feeding advice from healthcare providers and grandmothers during the first years of life.

Even though the present review aimed to explore parental feeding experiences of both genders, it was observed that mothers represent the vast majority of research participants. This was not surprising, as fathers’ underrepresentation in research on childhood dietary behaviours has been highlighted in the past^(89)^. The male-derived data included in the present review indicated that fathers played an assisting role during complementary feeding whilst allowing their female partners to lead in the decision-making process regarding timing of introduction and type of complementary foods. The quantitative literature that looked at both parents’ feeding practices also suggests that mothers and fathers have distinct interaction with children regarding food with fathers being less likely to monitor children’s food intake and apply restrictions on food compared with mothers^(90)^. In general, literature on fathers’ feeding practices is limited and focuses primarily on childhood nutrition as opposed to infancy. However, mealtime interactions between fathers and children are important and fathers can be influential role models of dietary behaviours, as observational studies demonstrate^(91,92)^. Further qualitative studies would be greatly valuable in elucidating paternal perceptions of their role in shaping their children’s dietary behaviours during the first years of life.

### Critical appraisal of included studies

This systematic review also highlights areas lacking information within the included qualitative literature with the main ones being the limitations of findings and authors’ conflicts of interest. Reporting on the limitations enables other researchers to critically appraise the trustworthiness of study findings; as for including a declaration of competing interests, it clarifies whether there are potential sources of influence on study conduct and conclusions. Inclusion of these elements therefore contributes to the clarity and completeness of reporting. Overall, adhering to standards of reporting can contribute to the clarity of qualitative manuscripts and help their authors disseminate them successfully.

### Limitations

Like in all systematic reviews of qualitative evidence, the final synthesis is prone to vary between researchers and to be driven by their individual research priorities and scientific background^(93)^. In order to deal with this, great care went to achieve a methodological interpretation and aggregation of study findings, and the use of an explicit protocol offered transparency to this process. Additionally, alongside the first author, two additional reviewers monitored the synthesis process and offered guidance.

The majority of qualitative research on parental feeding practices during complementary feeding currently comes from the UK, USA and Australia. Hence, the views of parents living in other upper-middle- and high-income countries, particularly in Europe and Asia, were underrepresented limiting the generalisability of the findings of this systematic review. Moreover, the information available on the socio-economic and family status of studies’ participants was limited. As a result, it was difficult to differentiate between feeding experiences of parents living in distinct social and family circumstances.

When reading this review, one also needs to consider that the feeding practices may be falsely self-reported if parents feel that they are being evaluated by the researcher. For instance, parents may disguise their poor compliance with feeding guidelines, which can become a barrier to an in-depth exploration of the underlying factors of poor compliance with the guidelines.

Despite its limitations, the process of undertaking this systematic review was characterised by transparency with its protocol having been published before the literature search. This paper was also structured according to the PRISMA guidance to ensure clear presentation of design and findings^(24)^.

Two previous systematic reviews by Harrison *et al.* and Matvienko-Sikar *et al.* have explored parental perceptions of and experiences during complementary feeding^(18,19)^. The present paper, however, provides an updated review of the evidence and provides an in-depth exploration of topics that were not previously addressed (e.g. experiences of baby-led-weaning mothers, paternal involvement in complementary feeding and an overview of all factors that influence choice of complementary foods). Consequently, the additional studies that were incorporated in the present review contributed to a more comprehensive synthesis of the factors in relation to parental feeding practices.

## Conclusions and future recommendations

This systematic review of qualitative evidence provided a comprehensive report on factors that parents take into consideration during complementary feeding in upper-middle- and high-income countries. Early introduction of complementary feeding was often reported, and misinterpreting baby cues as readiness for solids was quoted as the main reason. Parents demonstrated poor understanding of the evidence base of the complementary feeding guidelines, which were perceived to be a one-size-fits-all approach. The choice of complementary foods was mainly based on the perceived health properties of foods, though there was often uncertainty as to which foods need to be avoided at what stage.

These findings indicate a number of factors that can be barriers to complying with the complementary feeding guidelines and therefore are pertinent to policy makers, researchers and health practitioners that aim to help parents improve their feeding practices. Committees involved in developing national guidelines and recommendations around complementary feeding need to be mindful of the value of practical and easy-to-understand guidelines that reflect current research and acknowledge the cultural background of caregivers living in that area. The complementary feeding recommendations for every country should be discussed with parents with flexibility and without disregarding their children’s unique characteristics and needs. Attention needs to be drawn to vulnerable groups of parents (teenage mums or mothers living remotely with limited access to healthcare staff) who, due to their personal circumstances, are more prone to seek and take on board advice that may be outdated or not evidence based. Widening complementary feeding education to include fathers and other family members (e.g. grandmothers) may help to increase mothers’ feeling of being supported in feeding their child by ensuring a supportive environment and reducing pressure to go against guidelines. Fathers’ role during the period of complementary feeding is currently under-researched.

## Supplementary material

For supplementary material accompanying this paper visit https://doi.org/10.1017/S1368980021001749. click here to view supplementary material
