# Supplementary material for: Parental considerations during complementary feeding in higher income countries: a systematic review of qualitative evidence
Source: Public Health Nutr. 2021 Apr 20;24(10):2834–47. doi: 10.1017/S1368980021001749 (PMC9884778; doi:10.1017/S1368980021001749)
Supplement: Supplementary file 1 [file S1368980021001749sup.zip › urn_cambridge.org_id_binary_20210514023811942-0211_S1368980021001749sup001.docx]

Table 1: Characteristics of included studies

Abbreviations: CF= complementary feeding, N/R= not reported, y=years, w = weeks, mo = months

^1^ Study recruited from clinics that serve low-income populations – not a specific inclusion criterion

| **Study No** | **Study ID** | **Country** | **Phenomenon of interest** | **Specific eligibility criteria** | **Caregivers’ gender**  **(F/M)** | **Caregivers’ age (y) (M**±**SD, range)** | **Children’s age (M**±**SD, range)** | **First-time parents (*N*)** | **Sample size (*N*)** | **Sampling method** | **Data collection approach** | **Data analysis approach** |
| --- | --- | --- | --- | --- | --- | --- | --- | --- | --- | --- | --- | --- |
|  | Abu Shosha 2020 | Jordan | CF practices of Jordanian mothers | Female,  who breastfed | F | 29 ± 5.3 | 14 mo | N/R | 35 | Purposive | Focus groups | Thematic |
|  | Anderson 2001 | UK | Attitudes around the timing of CF | Female,  delivered locally | F | 27 ± 4.8 | 13 ± 4 w  (8 – 18) | 22 | 29 | Purposive | Focus groups | Thematic |
|  | Anderson 2010 | USA | Fathers’ perceptions of their role in infant feeding | Male,  WIC income eligibility | M | 21 – 57 | 3 – 6 mo | 10 | 21 | Purposive, Snowball | Phone interviews | Thematic |
|  | Arden 2010 | UK | Factors influencing adherence to 6-mo recommendation | N/R | F | 32.3 ± 4.5  (22 – 45) | 80 ± 38 w  (26-155) | N/R | 105 | Purposive | Individual interviews  (via email) | Content |
|  | Arden 2015 | UK | Benefits & challenges of baby-led weaning (BLW) | BLW parents | F | 29 – 39 | 9 – 15 mo | 12 | 15 | Purposive | Individual interviews  (via email) | Thematic |
|  | Beck 2018 | USA | CF practices of low-income Latinos | Low income ^1^, Latino parents in San Francisco | F & M | 31.9 | 11.6 mo | N/R | 26 | Purposive | Individual interviews | General inductive |
|  | Begley 2019 | Australia | Mothers’ infant feeding knowledge & practices | Female with children 4-18 mo | F | M: 30  22 – 44 | M: 9.6 mo  (4 – 18) | N/R | 42 | Purposive | Focus groups | Thematic  (social constructionist) |
|  | Boak 2016 | Australia | Mothers’ experiences of infant feeding | Taking part in *Splash!* cohort | F | 20 – 43 | 4 – 15 mo | 11 | 32 | Purposive | Individual interviews | Thematic content |
|  | Bramhagen 2006 | Sweden | Parents’ experiences  of feeding situations & contact with the child health services | N/R | F | 20 – 42 | 0.8 – 1.3 y | 4 | 18 | Purposive | Individual interviews | Content |
|  | Brown 2013 | UK | Positive & negative experiences of BLW | Female,  BLW parents | F | 28.6 ± 5.6 | 12-18 mo | N/R | 36 | Purposive, Snowball | Individual interviews | Content/ Thematic |
| **Study No** | **Study ID** | **Country** | **Phenomenon of interest** | **Specific eligibility criteria** | **Caregivers’ gender**  **(F/M)** | **Caregivers’ age (y) (M**±**SD, range)** | **Children’s age (M**±**SD, range)** | **First-time parents (*N*)** | **Sample size (*N*)** | **Sampling method** | **Data collection approach** | **Data analysis approach** |
|  | Cameron 2012 | New Zealand | Healthcare staff’s & mothers’ knowledge of & attitudes to BLW | Female,  BLW parents | F | N/R | 8-24 mo | N/R | 20 | Purposive, Snowball | Individual interviews | Content |
|  | Caton 2011 | UK | Parents’ feeding practices & ways to encourage child’ vegetable intake | N/R | F | 28.5 ± 1.2  (20 – 36) | 58 ± 4 w (34 – 76) | N/R | 13 | Purposive | Individual interviews | Thematic |
|  | Chakona 2020 | South Africa | Mothers’ infant feeding practices & influential factors | Caregivers  with children  0–24 mo | F | 34.7 ± 11.7 | 16.3 ± 6 mo | N/R | 94 | Purposive | Focus groups | Content analysis |
| 1. 11 | Cheney 2019 | USA | Sociocultural factors influencing infant feeding practices | Low income, Latino | F | 18 – 40 | <2 y | N/R | 19 | Conveni-ence | Focus groups | Codebook analysis |
|  | Dogan 2019 | Turkey | Mothers’ CF experiences | Female with children 6-24 mo | F | 29 ± 5.5  (18 – 38) | 10 ± 3.4 mo (6 – 18) | 8 | 20 | N/R | Individual interviews | Inductive Content |
| 1. 12 | Dutta 2006 | Brazil, Jamaica, Mexico, Panama | Cross-cultural perceptions of mothers about complementary food | Low income | F | N/R | 6 – 24 mo | N/R | 79 | Random, Purposive ^2^ | Individual interviews | Thematic |
|  | Garcia 2019 | UK | Parents’ CF practices & sources of information | Caregivers  with children  4–12 mo living in N. Lanarkshire | F | N/R ^3^ | N/R ^3^ | N/R ^3^ | 21 | Purposive | Individual interviews | Thematic |
|  | Griauzde 2020 | USA | Factors influencing feeding practices incl. social media | Female,  Hispanic,  living in Detroit | F | N/R 3 ^3^ | 6 – 36 ^3^ | N/R | 19 | Purposive | Individual interviews | Directed Content |
| 1. 13 | Heinig 2006 | USA | Factors influencing infant feeding behaviours | Female,  WIC participants | F | 24.6 ± 6.4 ^4^  29.1 ± 5.8 | 7 ± 2 mo ^4^  7.6 ± 2 mo | 23 ^4^  34 | 64  (27-37)^4^ | Purposive | Focus groups | Thematic |
| 1. 14 | Horodynski 2007 | USA | Maternal knowledge of & attitudes to CF | Enrolled in Medicaid | F | 17 – 41 | 3 w – 12mo | 1/3 of sample | 23 | Purposive, Snowball | Focus groups | Content / Thematic |

^2^ Random sampling in Brazil and Mexico and purposive sampling in Jamaica and Panama

^3^ These characteristics were reported for the samples of the quantitative part of the studies (mixed-methods studies)

^4^ Samples split into English- and Spanish-speaking participants; characteristics given respectively

Table 1: Characteristics of included studies - Continued

| **Study No**  Table 1: Characteristics of included studies - Continued | **Study ID** | **Country** | **Phenomenon of interest** | **Specific eligibility criteria** | **Caregivers’ gender**  **(F/M)** | **Caregivers’ age (y) (M**±**SD, range)** | **Children’s age (M**±**SD, range)** | **First-time parents (*N*)** | **Sample size (*N*)** | **Sampling method** | **Data collection approach** | **Data analysis approach** |
| --- | --- | --- | --- | --- | --- | --- | --- | --- | --- | --- | --- | --- |
|  | Horodynski 2012 | USA | CF practices of Native American mothers | Female, Native American | F | 19 – 56 | 0 – 12 mo | N/R | 42 | N/R | Focus groups | Content / Thematic |
|  | Horodynski 2014 | USA | Factors influencing CF practices of low-income adolescent mothers | Female, Low-income, Adolescent, First-time mum | F | 15 – 19 | 0 – 12 mo | 16 | 16 | Purposive | Focus groups | Content / Thematic |
|  | Jessri 2015 | Canada | Barriers of following the CF guidelines among Middle Eastern mothers | Female, Middle Eastern | F | 25.5 ± 10.0 | 3 – 11 mo | N/R | 22 | Purposive, snowball | Focus groups | Thematic |
|  | Kavanagh 2010 | USA | CF practices that may  predispose children to obesity | WIC participants | F ^5^ | >18 | 0 – 6 mo | N/R | 109 | Purposive | Focus groups | Thematic, (Grounded Theory) |
|  | Lee 2015 | USA | Cultural beliefs influencing infant feeding | Chinese descent, New York resident | F | 21-40 | <12 mo | 11 | 22 | Snowball, Conveni-ence | Individual interviews | Thematic |
| 1. 20 | Leung 2017 | UK | Cultural beliefs influencing infant feeding | Female, Chinese ethnicity, London resident | F | M: 36 | <12 mo | 8 | 10 | Purposive | Individual interviews | N/R |
| 1. 21 | Lindsay 2008 | Brazil | CF practices among low-income Brazilian mothers | Female, Low-income, Enrolled in PACS/PSF | F | M: 28  19 – 49 | 12 – 36 mo | N/R | 41 | Purposive | Focus groups | Content / Thematic |
|  | McFarren 2020 | USA | CF practices and experiences among Latino mothers | Latino with overweight children 6-18mo | F | M: 30  22 – 41 | M: 11 mo  6 – 18 | 6 | 14 | Purposive | Individual interviews | Thematic |
|  | McNally 2020 | UK | Mothers’ response to & understanding of infants’ appetite cues | Female, living in North England | F | 33 ± 2.9 | 15 ± 3.8 mo | 3 | 11 | Purposive | Video-elicited interviews | Thematic (template) |
|  | Merriman 2013 | Ireland | Children’s lives in Ireland  ^5^ Males were also recruited in the study, the sample however consisted predominately of females (105/109)  ^6^ Both parents were interviewed in 71.3% of cases, mothers were considered as the primary caregiver in most of them  ^7^ Mean ages are given for primary and secondary caregivers respectively | Enrolled in *Growing Up in Ireland* study | F & M ^6^ | M: 32  M: 36 ^7^ | 9 – 11 mo | 41 | 122 | Purposive, Stratified | Individual interviews | Thematic |
| **Study No**  Table 1: Characteristics of included studies - Continued | **Study ID** | **Country** | **Phenomenon of interest** | **Specific eligibility criteria** | **Caregivers’ gender**  **(F/M)** | **Caregivers’ age (y) (M**±**SD, range)** | **Children’s age (M**±**SD, range)** | **First-time parents (*N*)** | **Sample size (*N*)** | **Sampling method** | **Data collection approach** | **Data analysis approach** |
|  | Monterrosa 2012 | Mexico | Maternal CF behaviours | Female | F | 25.9 ± 5.3  (18 – 37) | 12.4 ± 4 mo (5 – 18) | N/R | 29 | Strategic, Conveni-ence | Individual interviews | Thematic |
| 1. 24 | Nielsen 2013 | Denmark | Maternal concerns & attitudes related to earlier and later CF | Female, Youngest child 7 or 13 mo | F | N/R | 7 & 13 mo | N/R | 45 | Purposive, Stratified, Snowball | Focus groups | Thematic |
| 1. 25 | Nielsen 2014 | Denmark | Maternal concerns & attitudes related to earlier and later CF | Female, Youngest child 7 or 13 mo | F | M:31  (21 – 41) | 7 & 13 mo | 25 | 45 | Purposive | Focus groups | Thematic |
| 1. 26 | Redsell 2010 | UK | Maternal CF practices and concerns | 3 sites with high risk of obesity and 1 with low | F ^8^ | 30.1 ± 6.28  (19 – 45) | M: 5.5 mo  (1 – 11) | N/R | 38 | Purposive | Focus groups | Thematic |
| 1. 27 | Rodriguez-Oliveros 2014 | Mexico | Classification, preparation & attributes of complementary foods | Female | F | M: 27.9 | < 2y | N/R | 12 | Purposive | Food-attributes exercises | Thematic |
| 1. 28 | Russel 2016 | Australia | Factors influencing feeding practices associated with a healthy weight gain | Female, No university degree | F | 29 ± 8  (21 – 38) | 7 ±4.5 mo  (2 – 11) | 15 | 29 | Purposive, Snowball | Phone interviews | Thematic |
| 1. 29 | Savage 2016 | USA | Parents’ CF beliefs & behaviours & sources of information | Female, WIC participants, Formula feeding | F | 25.6 ± 6.1  (19 – 43) | < 2y | N/R | 68 | Purposive | Focus groups & Phone interviews | Thematic |
| 1. 30 | Schwartz  2013 | France | Mothers’ CF practices & attitudes with a focus on vegetables | Female | F | 32.2 ± 4.0 (25.2–39.2) | 7.4 ± 3 mo  (3 – 14.3) ^9^ | 8 | 18 | Conveni-ence | Focus groups & Individual interviews | Thematic |
| 1. 31 | Spyreli 2019 | UK | Parents’ CF experiences | Infants 3-14 mo at recruitment | F | 30.3 ± 6  (19 – 39) | 7.7 ± 3 mo  (3 – 16) | 16 | 37 | Purposive, snowball | Focus groups | Thematic |
| ^8^ Males were also recruited in the study, the sample however consisted predominately of females (36/38)  ^9^ Children’s age originally reported in days (220.6 ± 96.9 days (89 – 429)  ^10^ Ages are reported separately for baby-led weaning mothers and those who followed a conventional approach respectively | Swanepoel 2019 | Australia | Mothers’ experiences with baby-led or conventional CF | Female,  Living in South-East Queensland | F | 29 ± 1.8 ^10^  35 ± 3.2 | N/R | 4 | 13 | Purposive | Focus groups (Photovoice) | Descriptive phenomeno-logical |
| **Study No** | **Study ID** | **Country** | **Phenomenon of interest** | **Specific eligibility criteria** | **Caregivers’ gender**  **(F/M)** | **Caregivers’ age (y) (M**±**SD, range)** | **Children’s age (M**±**SD, range)** | **First-time parents (*N*)** | **Sample size (*N*)** | **Sampling method** | **Data collection approach** | **Data analysis approach** |
| 1. 32 | Synnott 2007 | Germany, Italy, Spain, Scotland, Sweden | Parents’ CF practices | N/R | F ^11^ | N/R | <12 mo | 46 | 109 | Conveni-ence | Focus groups | Content / Thematic |
| 1. 33 | Thullen 2016 | USA | Co-parenting around infant feeding | Parenting dyads | F & M | N/R | 23 ± 11 mo | 8 | 24 dyads | Purposive | Individual interview | Thematic |
| 1. 34 | Tully 2019 | Island of Ireland | CF experiences and sources of guidance among low-income parents | Disadvantaged families | F ^11^ | M: 30  (16 – 47) | M: 8 mo  (3 – 18) | N/R | 83 | Purposive | Focus groups | Thematic |
| 1. 35 | Van der Merwe 2007 | South Africa | Mothers’ application & understanding of dietary guidelines for children | From all language groups | F | 20 – 30 (the majority) | 6 – 12 mo | N/R | 64 | Purposive | Focus groups | Thematic |
| 1. 36 | Walsh 2015 | Australia | Factors influencing first-time mothers’ decision making around introduction of solid foods | Female, First-time mum | F | N/R | 6 – 12 mo | 21 | 21 | Purposive, Conveni-ence | Focus groups & Individual interviews | Thematic (Theory Planned Behaviours) |
|  | Wrottesley 2020 | South Africa | Mothers’ infant feeding practices & influential factors | Sample of existing study ^12^ | F | 26 ± 4.3 ^13^  26 ± 4.5  28 ± 5.5 | 0 – 24 mo | 6 | 19 | Random | Focus groups & Individual interviews | Thematic |
| 1. 37 | Yue 2016 | China | Parents’ CF practices & factors that impede adherence to guidelines | N/R | F | N/R | 6 – 18 mo | N/R | 60 | Random | Individual interviews | Thematic |

^11^ Males were also recruited in the study, the sample however consisted predominately of females

^12^ The International Atomic Energy Agency multicentre infant body composition study

^13^ Mothers’ ages are reported separately for those with a child 0–6 months old, 7-14 months old and 15-24 months old respectively

Table 1: Characteristics of included studies - Continued

Table 2: List of studies excluded following full-text screening

| **Study ID** | **Primary reason for exclusion** | **More information** |
| --- | --- | --- |
| 1. Abel et al, 2001 | Year of study | Focus groups before 2001 |
| 1. Aidarous & Ahmad, 2016 | Full text not available |  |
| 1. Andrews et al, 2015 | Study design | Qualitative evaluation of intervention |
| 1. Babington, 2006 | Age of participants’ children | Lack of clarity in the paper – author uncontactable |
| 1. Babington, 2007 | Age of participants’ children | Birth – 6y |
| 1. Bentley et al, 2017 | Study doesn’t discuss weaning |  |
| 1. Brotanek et al, 2009 | Age of participants’ children | Birth – 4y |
| 1. Brown & Lee, 2011 | Focuses on breastfeeding |  |
| 1. Cameron et al, 2011 | Full text not available |  |
| 1. Carstairs et al, 2017 | Age of participants’ children | Birth – 4y |
| 1. Chen, 2010 | Study doesn’t discuss weaning |  |
| 1. Cheresheva, 2015 | Criteria RE participants not met | It is a presentation of pre-posted online narratives |
| 1. Cidro et al, 2014 | Study design | Qualitative evaluation of intervention |
| 1. Condon et al, 2015 | Participants not the primary caregivers | Mothers and grandmothers |
| 1. Cook et al, 2020 | Age of participants’ children | Birth – 5y |
| 1. Cricco-Lizza, 2006 | Focuses on breastfeeding |  |
| 1. Culhane-Pera et al, 2002 | Full text not available |  |
| 1. Dodgson et al, 2002 | Focuses on breastfeeding |  |
| 1. Du Plessis et al, 2018 | Study doesn’t discuss weaning |  |
| 1. Du Plessis et al, 2021 | Study doesn’t discuss weaning |  |
| 1. Ertem & Ergun, 2013 | Focuses on breastfeeding |  |
| 1. Escobar et al, 2018 | Full text not available in English |  |
| 1. Forero et al, 2018 | Full text not available in English |  |
| 1. Galegos et al, 2013 | Focuses on breastfeeding |  |
| 1. Gericke et al, 2010 | Full text not available |  |
| 1. Heinig et al, 2009 | Focuses on breastfeeding |  |
| 1. Heller et al, 2019 | Full text not available |  |
| 1. Helvey, 2012 | Study doesn’t discuss weaning |  |
| 1. Hilbig et al, 2012 | Focuses on breastfeeding |  |
| 1. Hoban & Liamputtong 2017 | Study doesn’t discuss weaning |  |
| 1. Hodges et al, 2008 | Study doesn’t discuss weaning |  |
| 1. Jama et al, 2018 | Children’s health status |  |
| 1. Jones, 2010 | Set in a developing country |  |
| 1. Kim et al, 2012 | Full text not available |  |
| 1. Kordsalarzeh et al, 2018 | Focuses on breastfeeding |  |
| 1. Kruger & Gericke, 2003 | Focuses on breastfeeding |  |
| 1. Kudlova, 2005 | Full text not available in English |  |
| 1. Kuswara et al, 2016 | Focuses on breastfeeding |  |
| 1. Lakhanpaul et al, 2020 | Participants not the primary caregivers |  |
| 1. Lakshman et al, 2012 | Study design | qualitative evaluation of intervention |
| 1. Liamputtong & Nakscook, 2001 | Year of study | Interviews before 2001 |
| 1. Lima et al, 2014 | Full text not available in English |  |
| 1. Lindsay et al, 2009 * | Study doesn’t discuss weaning |  |
| 1. Lindsay et al, 2017 | Age of participants’ children | 2 – 5y |
| 1. Lopez del Valle et al, 2005 | Age of participants’ children | Birth – 5y |
| 1. Lovelace & Rabiee-Khan, 2015 | Age of participants’ children | Birth – preschool age |
| 1. Majee et al, 2017 | Study doesn’t discuss weaning |  |
| 1. Maliwichi & Nesengani, 2013 | Full text not available |  |
| 1. Mangwane et al, 2010 | Full text not available |  |
| 1. Marshall et al, 2020 | Age of participants’ children | Birth – 5y |
| 1. Maslin et al, 2015 * | Children’s health status | Babies with cow’s milk allergy included |
| 1. McGarvey et al, 2006 | Study design | qualitative evaluation of intervention |
| 1. Mohamad et al, 2018 | Study design | Mixed methods, qualitative element not sufficient |
| 1. More, 2015 | Full text not available |  |
| 1. Murphy, 2007 | Study doesn’t discuss weaning |  |
| 1. Murray et al, 2008 | Focuses on breastfeeding |  |
| 1. Myers et al, 2014 | Age of participants’ children | Birth – 8y |
| 1. Quintero Romero et al, 2006 | Study design | Mixed methods – no significant qualitative element |
| 1. O’Key & Hugh-Jones, 2010 * | Age of participants’ children | Birth – 10y |
| 1. Peacock-Chambers et al, 2017 | Focuses on breastfeeding |  |
| 1. Raven et al, 2007 | Participants not the primary caregivers | Mothers, grandmothers and health workers |
| 1. Redsell et al, 2011 | Full text not available |  |
| 1. Rehayem et al, 2020 | Focuses on breastfeeding |  |
| 1. Rudzik & Ball, 2016 | Focuses on breastfeeding |  |
| 1. Sacco et al, 2007 | Study design | Cross-sectional |
| 1. Samli et al, 2006 | Full text not available in English |  |
| 1. Scott et al, 2009 | Age of participants’ children | Birth – 7y |
| 1. Spence et al, 2016 | Study design | qualitative evaluation of intervention |
| 1. Stapleton et al, 2009 | Study doesn’t discuss weaning |  |
| 1. Steinman et al, 2010 | Focuses on breastfeeding |  |
| 1. Tarrant et al, 2004 | Focuses on breastfeeding |  |
| 1. Valencia et al, 2016 | Study doesn’t discuss weaning |  |
| 1. Woo Baidal et al, 2015 | Age of participants’ children | Pregnant women also interviewed – findings reported together |
| 1. York & Hoban, 2013 | Age of participants’ children | Participants are pregnant women |

Table 2: List of studies excluded following full-text screening - Continued

| **Study ID**  Table 3: Comparison of all included studies against the 21 Standards for Reporting Qualitative Research - Green indicates that the study has sufficiently covered the standard, orange indicates inadequate information and red complete omission of information | **Title** | **Abstract** | **Introduction** | | **Methods** | | | | | | |
| --- | --- | --- | --- | --- | --- | --- | --- | --- | --- | --- | --- |
|  |  |  | **Problem**  **formulation** | **Research**  **question** | **Qualitative approach** | **Researcher**  **characteristics** | **Context** | **Sampling strategy** | **Ethical issues** | **Data collection** | **Data collection instruments** |
| 1. Abu Shosha 2020 | ✓ | ✓ | ✓ | ✓ | ✓ | X | ~ | ✓ | ✓ | ✓ | ✓ |
| 1. Anderson 2001 | ~ | ~ | ✓ | ✓ | ✓ | ✓ | ✓ | ~ | X | ~ | ~ |
| 1. Anderson 2010 | ~ | ✓ | ✓ | ✓ | X | ✓ | ~ | ✓ | ✓ | ~ | ✓ |
| 1. Arden, 2010 | ~ | ~ | ✓ | ✓ | ~ | X | X | ~ | ~ | ✓ | ✓ |
| 1. Arden 2015 | ~ | ✓ | ✓ | ✓ | ~ | ~ | X | ~ | ✓ | ✓ | ✓ |
| 1. Beck 2018 | ~ | ✓ | ✓ | ✓ | X | ~ | ✓ | ✓ | ✓ | ✓ | ✓ |
| 1. Begley 2019 | ✓ | ~ | ✓ | ✓ | ~ | ✓ | ✓ | ✓ | ✓ | ✓ | ✓ |
| 1. Boak 2016 | ✓ | ✓ | ✓ | ✓ | ✓ | ✓ | ✓ | ✓ | ✓ | ✓ | ✓ |
| 1. Bramhagen 2006 | ✓ | ✓ | ✓ | ✓ | ~ | ✓ | ✓ | ~ | ✓ | ~ | ~ |
| 1. Brown 2013 | ~ | ✓ | ✓ | ✓ | ~ | ~ | X | ✓ | ✓ | ~ | ✓ |
| 1. Cameron 2012 | ✓ | ✓ | ✓ | ✓ | ✓ | ~ | ~ | ✓ | ~ | ✓ | ✓ |
| 1. Caton 2011 | ✓ | ✓ | ✓ | ✓ | ✓ | ✓ | ✓ | ~ | X | ✓ | ✓ |
| 1. Chakona 2020 | ~ | ✓ | ✓ | ✓ | ~ | ~ | ✓ | ~ | ✓ | ✓ | ✓ |
| 1. Cheney 2019 | ~ | ~ | ✓ | ✓ | ✓ | ✓ | ✓ | ✓ | ✓ | ✓ | ✓ |
| 1. Dogan 2019 | ✓ | ~ | ✓ | ✓ | ~ | X | ~ | ~ | ~ | ~ | ~ |
| 1. Dutta 2006 | ~ | ✓ | ✓ | ✓ | ✓ | ~ | ✓ | ✓ | ✓ | ✓ | ~ |
| 1. Garcia 2019 | ~ | ✓ | ✓ | ✓ | ~ | X | ✓ | ✓ | ✓ | ✓ | ✓ |
| 1. Griauzde 2020 | ✓ | ~ | ✓ | ✓ | ✓ | X | ✓ | ✓ | ~ | ~ | ✓ |
| 1. Heinig 2006 | ~ | ~ | ✓ | ✓ | ✓ | ✓ | ~ | ✓ | ✓ | ✓ | ~ |
| 1. Horodynski 2007 | ~ | ✓ | ✓ | ✓ | ✓ | ✓ | ~ | ~ | ✓ | ~ | ✓ |
| 1. Horodynski 2012 | ~ | ~ | ✓ | ✓ | ~ | ✓ | ~ | X | ✓ | ✓ | ✓ |
| 1. Horodynski 2014 | ~ | ✓ | ✓ | ✓ | ✓ | ~ | ~ | ~ | ✓ | ✓ | ✓ |
| 1. Jessri 2015 | ✓ | ~ | ✓ | ✓ | ✓ | ✓ | ~ | ✓ | ✓ | ✓ | ~ |
| 1. Kavanagh 2010 | ~ | ~ | ✓ | ✓ | ✓ | ✓ | ~ | ~ | ✓ | ✓ | ~ |
| 1. Lee 2015 | ~ | ✓ | ✓ | ✓ | ✓ | X | ~ | ~ | ✓ | ✓ | X |
| 1. Leung 2017 | ~ | ✓ | ✓ | ✓ | ~ | ✓ | ✓ | ~ | X | ✓ | X |
| 1. Lindsay 2008 | ✓ | ✓ | ✓ | ✓ | ~ | ✓ | ✓ | ~ | ✓ | ✓ | ~ |
| 1. McFarren 2020 | ✓ | ✓ | ✓ | ✓ | ~ | X | ✓ | ✓ | ✓ | ~ | ✓ |
| 1. McNally 2020 | ~ | ✓ | ✓ | ✓ | ~ | X | ~ | ~ | ✓ | ✓ | ✓ |
| 1. Merriman 2013 | ✓ | ~ | ✓ | ✓ | ✓ | ~ | X | ✓ | ✓ | ✓ | ~ |
| **Study ID** | **Title** | **Abstract** | **Introduction** | | **Methods** | | | | | | |
|  |  |  | **Problem**  **formulation** | **Research**  **question** | **Qualitative approach** | **Researcher**  **characteristics** | **Context** | **Sampling strategy** | **Ethical issues** | **Data collection** | **Data collection instruments** |
| 1. Monterrosa 2012 | ~ | ~ | ✓ | ✓ | ✓ | ✓ | ✓ | ✓ | ✓ | ✓ | ~ |
| 1. Nielsen 2013 | ✓ | ~ | ✓ | ✓ | ✓ | ~ | ~ | ~ | ✓ | ✓ | ~ |
| 1. Nielsen 2014 | ~ | ✓ | ✓ | ✓ | ✓ | ✓ | ✓ | ~ | ✓ | ~ | ✓ |
| 1. Rodriguez-Oliveros 2014 | ~ | ✓ | ✓ | ✓ | ✓ | ✓ | ✓ | X | ✓ | ✓ | ~ |
| 1. Redsell 2010 | ~ | ✓ | ✓ | ✓ | X | ✓ | X | ~ | ✓ | ✓ | ✓ |
| 1. Russell 2016 | ✓ | ✓ | ✓ | ✓ | ✓ | ~ | ✓ | ✓ | ✓ | ✓ | ✓ |
| 1. Savage 2016 | ✓ | ~ | ✓ | ✓ | X | ✓ | ~ | ✓ | ✓ | ~ | ✓ |
| 1. Schwartz 2013 | ✓ | ✓ | ✓ | ✓ | ✓ | ✓ | ~ | ✓ | ~ | ✓ | ✓ |
| 1. Spyreli 2019 | ✓ | ✓ | ✓ | ✓ | ~ | ✓ | ~ | ✓ | ✓ | ✓ | ✓ |
| 1. Swanepoel 2019 | ~ | ✓ | ✓ | ✓ | ✓ | ✓ | ~ | ~ | ✓ | ✓ | ✓ |
| 1. Synnott 2007 | ✓ | ~ | ✓ | ✓ | ✓ | ✓ | ~ | ~ | ~ | ✓ | ~ |
| 1. Thullen 2016 | ~ | ✓ | ✓ | ✓ | ~ | X | ~ | ~ | ~ | ~ | ~ |
| 1. Tully 2019 | ~ | ✓ | ✓ | ✓ | ✓ | ✓ | ✓ | ✓ | ✓ | ✓ | ✓ |
| 1. Van der Merwe 2007 | ~ | ~ | ✓ | ✓ | ✓ | ~ | ✓ | ~ | ✓ | ✓ | ~ |
| 1. Walsh 2015 | ✓ | ✓ | ✓ | ✓ | ✓ | ~ | ~ | ✓ | ✓ | ~ | ~ |
| 1. Wrottesley 2020 | ~ | ~ | ✓ | ✓ | ~ | ✓ | ~ | ✓ | ✓ | ✓ | ✓ |
| 1. Yue 2016 | ✓ | ✓ | ✓ | ✓ | ✓ | ✓ | ✓ | ~ | ✓ | ✓ | ~ |

Table 3: Comparison of all included studies against the 21 Standards for Reporting Qualitative Research - Continued

| **Study ID**  Table 3: Comparison of all included studies against the 21 Standards for Reporting Qualitative Research - Continued | **Methods** | | | | | | **Results** | | **Discussion** | | **Conflicts of Interest** | **Funding** |
| --- | --- | --- | --- | --- | --- | --- | --- | --- | --- | --- | --- | --- |
|  | **Units of study** | **Data processing** | | **Data analysis** | | **Techniques enhancing credibility** | **Synthesis & Interpretation** | **Links to empirical data** | **Integration with prior work** | **Limitations** |  |  |
| 1. Abu Shosha 2020 | ✓ | ~ | | ✓ | | ✓ | ✓ | ✓ | ~ | ~ | ✓ | ✓ |
| 1. Anderson 2001 | ~ | ~ | | X | | ✓ | ✓ | ~ | ~ | ~ | X | ✓ |
| 1. Anderson 2010 | ✓ | ✓ | | ✓ | | ✓ | ✓ | ~ | ✓ | ✓ | ✓ | ✓ |
| 1. Arden 2010 | ✓ | ✓ | | ~ | | ✓ | ✓ | ✓ | ~ | ~ | ✓ | ✓ |
| 1. Arden 2015 | ✓ | ✓ | | ✓ | | X | ✓ | ✓ | ✓ | ✓ | X | ✓ |
| 1. Beck 2018 | ~ | ~ | | ✓ | | ✓ | ✓ | ✓ | ~ | ✓ | ✓ | ✓ |
| 1. Begley 2019 | ✓ | ✓ | | ✓ | | ✓ | ✓ | ✓ | ✓ | ✓ | ✓ | ✓ |
| 1. Boak 2016 | ✓ | ✓ | | ✓ | | ✓ | ✓ | ✓ | ✓ | ✓ | X | ✓ |
| 1. Bramhagen 2006 | ✓ | ✓ | | ✓ | | ✓ | ✓ | ✓ | ~ | ✓ | X | ✓ |
| 1. Brown 2013 | ~ | ~ | | ✓ | | ✓ | ✓ | ✓ | ~ | X | ✓ | ✓ |
| 1. Cameron 2012 | X | ~ | | ✓ | | ✓ | ✓ | ✓ | ~ | ✓ | ✓ | ✓ |
| 1. Caton 2011 | ✓ | ✓ | | ✓ | | ✓ | ✓ | ✓ | ~ | X | X | X |
| 1. Chacona 2020 | ✓ | ~ | | ✓ | | X | ✓ | ✓ | ✓ | ✓ | ✓ | ✓ |
| 1. Cheney 2019 | ~ | ~ | | ✓ | | ✓ | ✓ | ✓ | ✓ | ~ | ✓ | ✓ |
| 1. Dogan 2019 | ✓ | ~ | | ✓ | | ✓ | ✓ | ✓ | ~ | X | ✓ | ✓ |
| 1. Dutta 2006 | X | ✓ | | ✓ | | ✓ | ✓ | ~ | ~ | X | X | ✓ |
| 1. Garcia 2019 | ~ | ~ | | ~ | | X | ✓ | ✓ | ✓ | ✓ | ✓ | X |
| 1. Griauzde 2020 | ✓ | ~ | | ✓ | | ✓ | ✓ | ✓ | ✓ | ✓ | ✓ | ✓ |
| 1. Heinig 2006 | ✓ | ✓ | | ✓ | | ✓ | ✓ | ✓ | ~ | ~ | ✓ | ✓ |
| 1. Horodynski 2007 | ~ | ~ | | ✓ | | ✓ | ✓ | ✓ | ~ | ✓ | X | ✓ |
| 1. Horodynski 2012 | ~ | ~ | | ✓ | | ✓ | ✓ | ✓ | ~ | ~ | X | ✓ |
| 1. Horodynski 2014 | ~ | ~ | | ✓ | | X | ~ | ✓ | ~ | ~ | X | ✓ |
| 1. Jessri 2015 | ~ | ✓ | | ✓ | | ✓ | ✓ | ✓ | ✓ | ✓ | ✓ | ✓ |
| 1. Kavanagh 2010 | ~ | ✓ | | ✓ | | ✓ | ✓ | ✓ | ~ | X | ✓ | ✓ |
| 1. Lee 2015 | ~ | ~ | | ✓ | | X | ✓ | ✓ | ✓ | ~ | ✓ | X |
| 1. Leung 2017 | ~ | ~ | | X | | X | ✓ | ✓ | ~ | ~ | X | X |
| 1. Lindsay 2008 | ✓ | ✓ | | ✓ | | ✓ | ✓ | ✓ | ~ | X | X | ✓ |
| 1. McFarren 2020 | ✓ | ~ | | ~ | | ✓ | ✓ | ✓ | ✓ | ✓ | ✓ | ✓ |
| 1. McNally 2020 | ✓ | ~ | | ✓ | | ✓ | ✓ | ✓ | ✓ | ✓ | ✓ | ✓ |
| 1. Merriman | ✓ | ~ | | ✓ | | X | ✓ | ✓ | ~ | X | X | ✓ |
| **Study ID** | **Methods** | | | | | | **Results** |  | **Discussion** |  | **Conflicts of Interest** | **Funding** |
|  | **Units of study** | | **Data processing** | | **Data analysis** | **Techniques enhancing credibility** | **Synthesis & Interpretation** | **Links to empirical data** | **Integration with prior work** | **Limitations** |  |  |
| 1. Monterrosa 2012 | ~ | ~ | | ✓ | | ✓ | ✓ | ✓ | ✓ | ✓ | X | ✓ |
| 1. Nielsen 2013 | ~ | ✓ | | ✓ | | ✓ | ✓ | ✓ | ✓ | ~ | ✓ | ✓ |
| 1. Nielsen 2014 | ✓ | ~ | | ✓ | | X | ✓ | ✓ | ✓ | ~ | X | ✓ |
| 1. Rodriguez-Oliveros 2014 | ✓ | ~ | | ✓ | | ✓ | ✓ | ✓ | ✓ | ✓ | ✓ | ✓ |
| 1. Redsell 2010 | ~ | ✓ | | ✓ | | ✓ | ✓ | ✓ | ~ | ✓ | ✓ | ✓ |
| 1. Russell 2016 | ✓ | ✓ | | ✓ | | ✓ | ✓ | ✓ | ✓ | ~ | ✓ | ✓ |
| 1. Savage 2016 | ✓ | ~ | | ✓ | | ✓ | ✓ | ✓ | ~ | ~ | X | ✓ |
| 1. Schwartz 2013 | ~ | ~ | | ✓ | | ✓ | ✓ | ✓ | ~ | ✓ | X | ✓ |
| 1. Spyreli 2019 | ✓ | ✓ | | ✓ | | ✓ | ✓ | ✓ | ✓ | ✓ | ✓ | ✓ |
| 1. Swanepoel 2019 | ✓ | ✓ | | ✓ | | ✓ | ✓ | ✓ | ✓ | ✓ | ✓ | ✓ |
| 1. Synnott 2007 | ~ | ~ | | ✓ | | ✓ | ✓ | ✓ | ~ | ✓ | X | ✓ |
| 1. Thullen 2016 | ~ | ~ | | ✓ | | X | ✓ | ✓ | ~ | ~ | X | X |
| 1. Tully 2019 | ~ | ~ | | ✓ | | ✓ | ✓ | ✓ | ✓ | ✓ | ✓ | ✓ |
| 1. Van der Merwe 2007 | ~ | ~ | | ✓ | | ✓ | ✓ | X | ✓ | ~ | X | ✓ |
| 1. Walsh 2015 | ~ | X | | ✓ | | X | ✓ | ✓ | ✓ | ✓ | ✓ | ✓ |
| 1. Wrottesley 2020 | ✓ | ✓ | | ✓ | | ✓ | ✓ | ✓ | ✓ | ✓ | ✓ | ✓ |
| 1. Yue 2016 | ~ | ✓ | | ✓ | | X | ✓ | ✓ | ~ | ~ | X | ✓ |

Table 3: Comparison of all included studies against the 21 Standards for Reporting Qualitative Research - Continued

Table 4: Occurrence of topics within selected literature (N=47) – a priori selected topics and newly emerging topics

^1^ This can be further broken down into subcategories:
 baby being hungry (*N*=20); interest in food (*N*=15); changes in baby’s weight (*N*=10); developmental cues (*N*=9)

| Themes and subthemes | Number of studies | Proportion to all included studies |
| --- | --- | --- |
| *A priori selected topics:* |  |  |
| Factors that influence the choice of foods | **34** | **72%** |
| Actual/perceived health properties | 29 |  |
| Baby’s preferences/aversions | 13 |  |
| Cultural and religious beliefs | 13 |  |
| Food cost and availability | 9 |  |
| Parental factors (risk aversions, skills) | 18 |  |
| Knowledge of and attitudes towards WHO guidelines on timing of weaning | **22** | **47%** |
| Awareness of the recommended weaning age | 12 |  |
| Understanding the evidence basis of the guidelines | 7 |  |
| Attitudes towards the guidelines | 17 |  |
| Views on the available sources of complementary feeding advice | **38** | **81%** |
| Grandmothers | 25 |  |
| Health care professionals | 32 |  |
| Peer influence | 24 |  |
| Previous experience | 16 |  |
| Strategies to establish healthy eating habits | **17** | **36%** |
| Providing variety of foods | 11 |  |
| Repeated exposure to foods | 6 |  |
| Baby-led weaning | 5 |  |
| Modelling | 3 |  |
| Perceptions of commercial infant foods | **16** | **34%** |
| Additives and harmful ingredients | 9 |  |
| Poor taste | 4 |  |
| Inadequate nutritional value | 6 |  |
| Cost | 4 |  |
| *Newly emerging topics:* |  |  |
| Prompts for the introduction of solid foods | **27** | **57%** |
| Baby-related prompts^1^ | 25 |  |
| Mother-related prompts | 9 |  |
| Experiences of baby-led-weaning mothers | **6** | **13%** |
| Positive experiences | 5 |  |
| Negative aspects | 5 |  |
| Fathers’ role during weaning | **6** | **13%** |
| Feedback from mothers for improved weaning education | **18** | **38%** |
| Suggested areas of inadequate information | 13 |  |
| Health care professionals’ role in weaning education | 9 |  |
